# Interplay between sexual excitation and inhibition: impact on sexual function and neural correlates of erotic stimulus processing in women

**DOI:** 10.3389/fnbeh.2024.1386006

**Published:** 2024-05-15

**Authors:** Norina M. Schmidt, Juergen Hennig, Aisha J. L. Munk

**Affiliations:** Department of Differential and Biological Psychology, Justus-Liebig-University Giessen, Giessen, Germany

**Keywords:** erotic stimulus processing, sexual function, sexual excitation, sexual inhibition, dual control model, LPP amplitudes, oral contraceptives, event-related potentials

## Abstract

**Background:**

As outlined by the dual control model (DCM), individual differences in the regulation of sexual arousal following sexual stimulation depend on two distinct neurophysiological processes: sexual excitation (SE) and sexual inhibition (SI). Although associations with sexual function, behavior, and cue processing have been demonstrated in previous research, underlying neural correlates remain insufficiently explored. Moreover, interactive effects of SE/SI as proposed by the DCM, as well as factors impacting SE/SI properties, such as the use of oral contraceptives (OCs), have not received adequate attention in existing research.

**Methods:**

90 healthy, sexually active women (*n* = 51 using OCs, *n* = 39 naturally cycling) completed an Emotional-Picture-Stroop-Paradigm (EPSP) while a 64-channel EEG was recorded. LPP amplitudes toward erotic and neutral stimuli were consecutively computed as a marker of motivational salience and approach motivation. Additionally, women provided self-reports of SE/SI and sexual function. Moderation analyses were performed to assess interactive effects of SE/SI in predicting LPP amplitudes and sexual function.

**Results:**

Sexual function was negatively associated with SI levels but unrelated to SE. Higher SI was associated with reduced LPP amplitudes in response to erotic stimuli. This negative association was, however, attenuated for women high in SE, suggesting interactive effects of SE/SI. Furthermore, women using OCs reported lower SE compared to naturally cycling women.

**Conclusion:**

The observed findings provide additional psychophysiological evidence supporting the DCM and underscore the relevance of interactive SE/SI effects in stimulus processing and approach motivation. They also highlight the possible impact of OCs on psychosexual variables that warrants further research.

## 1 Introduction

Sexual desire can be elicited by various internal and external situational cues, such as sexual fantasies, physical closeness, the touch of an attractive partner, or romantic settings. It is not the situation alone, however, that determines sexual responses. Individuals exhibit stable interindividual differences in their responsiveness to these situational cues. The degree to which an individual is responsive to erotic cues is referred to as sexual excitation (SE). However, emerging sexual desire can also be dampened by factors like self-consciousness, concerns about one’s own sexual performance or possible risks of pregnancy and sexually transmitted diseases (STDs), along with “not-just-right”-feelings, or lack of interpersonal trust. Again, individuals vary in the extent to which they are bothered by these inhibiting situational aspects – a concept referred to as sexual inhibition (SI). According to the dual control model (DCM) (Bancroft and Janssen, 2000; Janssen and Bancroft, 2007; Bancroft et al., 2009), excitatory and inhibitory forces operate independently from each other, and adaptive sexual responses are based on a balanced interplay between these dynamics. Correspondingly, SE and SI have been considered as the gas pedal and brake of sexual arousal, respectively (Janssen and Bancroft, 2023). Affirming the theoretical assumption of independent forces, self-reported propensities for SE and SI have been shown to be either uncorrelated (Janssen et al., 2002a; Quinta Gomes et al., 2018), or to express only small positive or negative correlations (Graham et al., 2006; Velten et al., 2016a).

In laboratory studies, associations between self-report measures of SE/SI and subjective, as well as bodily reactions to erotic stimulation have been reported. Results are, however, heterogenous. In one of the first studies, Janssen et al. (2002b) reported higher genital and subjective sexual arousal to an erotic film clip in males high vs. low in SE. Furthermore, males experiencing less threat in association with sexuality, which can be understood as a facet of low SI, had stronger genital responses to a film clip indicating coercive sexual interactions. In a later study in a gender-mixed sample, Hodgson et al. (2016) reported significant associations between SE/SI and genital and subjective sexual arousal solely in women. While the findings regarding genital arousal supported the DCM framework, i.e., higher genital arousal was associated with higher SE and lower SI, results regarding subjective arousal were contradictory. Subjective arousal was unrelated to SI and negatively associated with SE. The authors argued that women high in SE might have more extensive experience with erotic content and thus perceive used film clips as less stimulating. Other studies found no associations between SE/SI and genital or subjective sexual arousal but reported higher sexual concordance, i.e., increased coherence between genital and subjective sexual arousal, in females with higher SE levels. Individuals high in SE might show greater sensitivity to bodily changes and thus perceive sexual arousal more precisely (Clifton et al., 2015). These laboratory studies used devices such as thermographic cameras, or vaginal photoplethysmography to measure genital arousal. The invasive nature of such techniques in a laboratory setting might however, be perceived as uncomfortable and shameful, therefore altering bodily and subjective arousal processes, especially in individuals prone to SI. Using self-reports of habitual sexual function (SF) and assessing neural, rather than genital, correlates of erotic stimulus processing thus constitutes a valuable addition to previous research. Self-report instruments assess regular, in contrast to laboratory, patterns of sexual behavior and arousal, including subjective arousal, lubrication, and frequency of orgasms (Rosen et al., 2000) and assessment of neural correlates associated with erotic stimulus processing is less invasive compared to thermographic cameras or vaginal photoplethysmography. It is, therefore, less likely to cause shame and discomfort in participants.

It is also a suitable approach to examine whether differential perception and processing of erotic stimuli underlie previously observed differential subjective and genital reactions in individuals with varying propensities for SE and SI. According to cognitive and information processing models, attending to and processing of sexual cues is necessary for sexual arousal and desire to occur (Janssen et al., 2000; Jong, 2009; Tavares et al., 2020). Thus, to gain a deeper understanding of SE and SI and resolve inconsistencies, it could be insightful to explore underlying processes, such as attention allocation and approach motivation.

So far, studies on attentional processes have centered on SE, as this DCM dimension is more strongly associated with processing of external sexual information (Janssen and Bancroft, 2023). Initial evidence revealed associations between SE and target detection time, as a measure of attentional engagement, in a dot probe task (Prause et al., 2008). Participants high in SE required more time to detect a target that appeared under a sexual vs. neutral image. Diverging interpretations were discussed by the authors: participants low in SE might experience higher levels of novelty when presented with erotic stimulus material, leading to increased attention allocation and faster reaction times. Participants high in SE, in turn, might require more time to disengage from sexual stimuli displayed before the dot, resulting in slower reaction times (Prause et al., 2008). However, in a later study, using a letter discrimination task with erotic stimuli as distractors, no effect of SE on attention was noted (Carvalho et al., 2018). Both studies examined mixed samples, consisting of approximately 50% males and females, and did not consider SI. In a recent study using an event-related potential (ERP) approach, Aguiar et al. (2023), however, reported associations between threat of performance failure, a facet of SI, and early automatic attentional processing of romantic and erotic stimulus material. Participants with a high propensity for SI showed elevated N200 amplitudes and reduced N200 latencies in response to these stimuli, suggesting amplified automatic processing. The N200 is an ERP component associated with novelty detection, visual attention and cognitive control (Folstein and van Petten, 2008). The authors argued that individuals worrying extensively about sexual performance could regard intimate stimuli as intimidating, leading to enhanced initial processing. SE was not associated with early stimulus processing (P100, P200, N200). Importantly, the study aimed to examine possible mediating effects of SE/SI in the relationship between neuroticism and erotic stimulus processing. Results indicated a complete mediation of the association between neuroticism and stimulus processing by SI (Aguiar et al., 2023).

To enhance our understanding of the attentional and motivational dynamics underlying SE/SI, the use of ERPs constitutes a particularly well-suited and advantageous technique. The high temporal resolution of ERP components and their well-described associations with specific cognitive and emotional processes (Hajcak et al., 2010), including those associated with erotic stimulus processing (Huberman et al., 2023), allow for the specific identification of different phases of stimulus processing affected by tendencies for SE/SI. So far, however, the study from Aguiar et al. (2023) was the only one to apply an ERP-based approach to assess neural correlates of the DCM framework. As early, attentional stages of stimulus processing (≤300 ms) were the focus, later ERPs, such as the late positive potential (LPP), which begins approximately 400 ms after stimulus onset and persists for several hundred milliseconds (Hajcak and Olvet, 2008), were not examined. Generally, the LPP is regarded as a neurophysiological marker of motivated attention, motivational salience and approach motivation (Ferrari et al., 2008; Gable and Harmon-Jones, 2013) and is associated with reward processing (Meadows et al., 2016). These psychological functions have previously been found to be associated with SE/SI (Prause et al., 2008; Gregory et al., 2015; Unterhorst et al., 2018; Turner et al., 2019). Additionally, the LPP has been shown to be especially sensitive to erotic content, with higher LPP amplitudes observed for erotic compared to neutral and, moreover, other emotional stimuli (van Lankveld and Smulders, 2008; Schmidt et al., 2022). Therefore, it might be a particularly suitable marker for studying neural correlates of SE/SI effects.

With regard to approach motivation, for instance, behavioral tasks suggest differential associations with SE and SI. Turner et al. (2019) conducted an implicit approach-avoidance task in a heterosexual male sample. SE predicted heightened approach bias (i.e., shorter reaction times in pull vs. push trials) to pictures of nude women. Threat of performance failure (SI), in turn, predicted an elevated avoidance bias toward these stimuli. In an fMRI study conducted in a female sample, SE was also found to be associated with greater reactivity to sexual cues in the ventral tegmental area (VTA), a key region in reward processing. SI in turn, was not associated with VTA activity (Gregory et al., 2015). Unterhorst et al. (2018) examined a greater variety of brain regions and reported positive associations between SI and activity in the anterior insula and prefrontal areas. SE was associated with activity in brain areas known to be involved in erotic stimulus processing, including the cerebellum, the inferior parietal lobules, the anterior insula, the dorsal anterior cingulate gyrus and the dorsal striatum.

In summary, previous research has highlighted the relevance of SE regarding attentional and motivational processes associated with erotic stimulus processing (Prause et al., 2008; Gregory et al., 2015). However, recent evidence also strengthens the importance of SI (Unterhorst et al., 2018; Turner et al., 2019; Aguiar et al., 2023). Notably, the DCM suggests that sexual arousal and behavior cannot be fully understood without considering the dynamic interplay of the SE/SI system – something that has not systematically been done in prior research, which predominantly focused on the independent impact of both forces (Janssen and Bancroft, 2023). Results of an erotic film clip study, conducted by Velten et al. (2016c), nevertheless highlighted the importance of considering interactive effects. They reported that for low levels of SI, SE positively predicted genital sexual arousal in women. For high SI, however, the pattern was reversed, with SE negatively predicting sexual arousal. Importantly, neither SE nor SI had direct effects on genital arousal. The authors argued that high levels of both dimensions could lead to inner conflicts reducing genital arousal. Importantly, such interference effects can only be examined when SE and SI are analyzed simultaneously. Further studies in this field are highly warranted, particularly given the consistent associations of SE and SI with risky sexual behavior and sexual dysfunctions. According to the DCM, these problems can emerge when the balanced interplay between SE and SI is disturbed and either of these forces is diminished or exaggerated (Bancroft et al., 2009). Heightened excitatory properties, which outweigh weak inhibitory processes, increase susceptibility to risky sexual behavior or sexual impulsivity. Correspondingly, higher SE has been shown to predict younger age at first sexual intercourse, higher number of intercourse partners and one-night stands as well as condom abandonment. Higher SI, in turn, showed reversed patterns with these risk behaviors (Velten et al., 2016b; Granados et al., 2017). On the contrary, an overactivation of inhibitory processes accompanied by low excitatory dynamics might predispose individuals to experience sexual dysfunctions (Bancroft et al., 2009). Velten et al. (2019a), for instance, examined a sample of 964 couples and observed consistent results in both genders: Higher SE related to higher sexual function (SF), while a negative association emerged for SI. In clinical samples, erectile dysfunctions in males and orgasm difficulties in females were associated with higher SI levels but unrelated to SE (Moura et al., 2020; Quinta-Gomes et al., 2022). Neural activity patterns in women diagnosed with hyposexual desire disorder (HSDD), however, support the dual process approach. In their study, Bianchi-Demicheli et al. (2011) reported diminished reward-related processing of erotic stimuli alongside heightened higher-order processing in parietal, frontal, and extrastriate cortices among HSDD patients as compared to healthy controls.

To sum up, research on associations of SE/SI with erotic stimulus processing and SF is highly pertinent but currently limited. Given the reported associations between SE/SI and clinically relevant phenomena such as risky sexual behavior and sexual dysfunctions, gaining a more profound understanding of underlying processes could offer valuable insights for informing treatment strategies. Based on the theoretic framework of the DCM, it is crucial to pay special attention to interactive effects of SE/SI as opposed to independent contributions. Given that diverging association patterns between SE/SI and sexual arousal have been described in male vs. female samples (Clifton et al., 2015; Hodgson et al., 2016; Janssen and Bancroft, 2023), gender-mixed samples could mask complex SE/SI interactions and render it difficult to correctly identify them. Importantly, gender differences have been reported for SE and SI properties, with higher SE levels in males and higher SI levels in females (Carpenter et al., 2008). The dynamic interplay of SE and SI might, therefore, depend on the relative strength of each force. In this regard, it is crucial to further consider factors that could systematically impact SE/SI properties. Although SE/SI are trait-dimensions that show substantial temporal stability, factors such as change in partnership status or SF have been shown to impact SE/SI scales (Velten et al., 2019b). Still, use of oral contraceptives (OCs), as an important factor known to impact female sexuality, has not previously been considered in SE/SI research. Yet, numerous studies indicate reduced SF and sexual desire (Wallwiener et al., 2010; Pastor et al., 2013; Huang et al., 2020) as well as reduced neural (Abler et al., 2013; Monciunskaite et al., 2019) and genital (Handy et al., 2023) reactivity toward erotic stimulus material in OC users. These aspects show strong overlap with SE as this dimension indicates responsiveness to erotic cues (Bancroft et al., 2009). If OC use affected SE, it might also alter the dynamic interplay of SE/SI, leading to diverging patterns of associations with SF and erotic stimulus processing in users vs. non-users. Although prevalence and comorbidity of sexual dysfunctions are higher in women compared to men (Laumann et al., 1999; Rosen, 2000; McCabe et al., 2016), research on sexuality and erotic stimulus processing has predominantly been focusing on male samples or gender differences, and studies exclusively examining the neural correlates of erotic stimulus processing in women are sparse (Ziogas et al., 2023). In light of this gap, the present study focuses on a female sample comprising healthy naturally cycling (NC) and OC using women.

Based on previously reported open questions, this study pursued three primary objectives:

(1) To assess neural correlates of SE/SI properties during erotic stimulus processing in females using ERP techniques.
(2) To examine interactive, as opposed to independent, effects of SE/SI on self-reported SF and neural correlates of erotic stimulus processing.
(3) To exploratorily examine the modulatory influences of OC use on SE/SI properties and SE/SI interactions.

Our specific expectations were as follows:

(1) SI and SE would demonstrate interactive effects in predicting a) self-reported SF and b) LPP amplitudes toward erotic stimuli.
(2) OC users would report lower SE compared to NC women.

We also aimed to exploratorily examine whether OC use moderates the association between SE/SI and (a) self-reported SF and (b) LPP amplitudes. Given the absence of prior research in this field, we did not have strong a-priori hypotheses regarding the direction of the SE/SI interaction effect or the moderating role of OC use.

## 2 Materials and methods

### 2.1 Study design

Data was collected as part of a comprehensive research project that examined the association of menstrual cycle phases, OC use, and personality traits with emotional stimulus processing in women. Participants in the study either had natural menstrual cycles or were using OCs. Results regarding these group comparisons have been reported elsewhere (Schmidt et al., 2022). As part of this research project, women were assessed on three measurement occasions corresponding to distinctive cycle phases (follicular phase, ovulatory phase, luteal phase in NC women; first and second week of OC treatment as well as OC break in OC users). Order of phases was randomized. On each measurement occasion, women completed an Emotional Picture Stroop Paradigm (EPSP) while an EEG was recorded, and ERPs were subsequently analyzed. For the current research question, only ERP data collected during the first measurement occasion were considered to ensure novelty of used stimulus material and exclude habituation effects.

### 2.2 Sample characteristics

Women were recruited via circular mails and flyers distributed at the Justus-Liebig-University in Giessen. After communication of initial interest, eligibility criteria were clarified in a standardized questionnaire- and telephone-based screening. Following criteria were checked: (1) age between 18 and 35, (2) nulliparous, (3) heterosexual, (4) absence of any physical or psychological health condition, (5) no intake of medication with central nervous system (CNS) or endocrine effects, (6) non-smoker, 7) right-handedness, (8) BMI > 18 < 26 kg/m^2^, (9) normal or corrected-to-normal vision and intact color vision. Women had either used combined OCs for at least 6 months in a 21/7 regimen or had not used any form of hormonal contraception for the same period of time. For the latter group, a regular menstrual cycle with a mean cycle duration of 26–30 days was mandatory.

A total of *N* = 117 female participants completed data assessment. *n* = 3 participants were excluded due to insufficient EEG data quality. Of the remaining *n* = 114 participants, *n* = 57 were NC, *n* = 57 were using combined OCs. Of these, *n* = 6 OC users and *n* = 18 NC women reported to not have had sexual intercourse within the past month. Due to the significantly higher proportion of NC women reporting absence of sexual intercourse, χ^2^_(1)_ = 7.60, *p* = 0.006, analyses were limited to *n* = 90 women (*n* = 51 using OCs, *n* = 39 naturally cycling) who reported sexual intercourse. This was done to avoid confounding of OC status and sexual activity, as former research indicated associations of LPP amplitudes and number of sexual intercourse partners as an indicator of sexual novelty/deprivation (Prause et al., 2015). Furthermore, the FSFI total score does not hold valid information for those without intercourse (Berner et al., 2004). Women without sexual activity within the past month reported higher SI, *t*_(112)_ = 2.65, *p* = 0.009, *d* = 0.61, but did not differ from sexually active women regarding SE, *t*_(112)_ = −0.65, *p* = 0.517. Of the sexually active women, *n* = 15 NC women were assessed during the follicular phase, *n* = 10 during ovulation and *n* = 14 during the luteal phase. OC users were assessed during the first week of a new OC blister (*n* = 24), during the second week (*n* = 15) and during the OC break (*n* = 12). Mean age in the sample was 22.64 years (SD = 2.07), NC and OC women did not differ significantly regarding age, *t*_(88)_ = 1.86, *p* = 0.067, although there was a tendency for NC women (*M ± SD*: 23.10 ± 2.22) to be slightly older than OC women (*M* ± *SD*: 22.29 ± 1.90). *N* = 80 women reported to be in a relationship, whereas *n* = 10 reported being single. Proportion of single and partnered women did not differ between OC and NC women, χ^2^_(1)_ = 0.20, *p* = 0.652.

Participants were compensated with 10 €/h or research participation credit. Written informed consent was obtained prior to assessment. The study was approved by the local ethics committee of the University of Giessen, Department of Psychology (application number: 2018-0022) and was conducted in agreement with the declaration of Helsinki.

### 2.3 Measures

SE/SI were assessed using the German version (Velten et al., 2016a) of the Sexual Excitation/Sexual Inhibition Inventory for Women (SESII-W) (Graham et al., 2006). It was included in an online survey assessing sociodemographic as well as personality characteristics. Participants were instructed to complete the survey prior to their last appointment. The SESII-W uses 23 and 13 items to assess SE and SI, respectively. These describe situational circumstances that might arouse (SE) or inhibit (SI) sexual arousal. Level of agreement to each statement is indicated on a scale of 1 (strongly disagree) to 4 (strongly agree). The SE factor is comprised of five second-order factors: Arousability, Sexual Power Dynamics, Setting, Partner Characteristics, and Smell. SI is comprised of the second-order factors Relationship Importance, Concerns about Sexual Function and Arousal Contingency. In the current sample, Cronbach’s alpha was α = 0.80 for SE and α = 0.71 for SI. Confirming the assumption of relatively independent forces, SE and SI showed only a small negative correlation with marginal significance, *r* = −0.21, *p* = 0.051.

SF was assessed using the German version (Berner et al., 2004) of the Female Sexual Function Index (FSFI) by Rosen et al. (2000) that was also included in the online survey package. The FSFI uses 19 items to assess six subcomponents of female SF, namely desire (two items), arousal, lubrication (each four items), orgasm, satisfaction and pain (each three items) which add up to a total score, with higher scores indicating higher SF. The score is indicative of SF in the past 4 weeks. For each item assessing sexual activity, a “no attempt of sexual activity/intercourse” answer category is given in which case the total score should not be interpreted (Berner et al., 2004). Answers are collected using five/six-point Likert scales. Cronbach’s alpha for the total score was α = 0.94.

### 2.4 Emotional picture stroop paradigm (EPSP)

**FIGURE 1:**
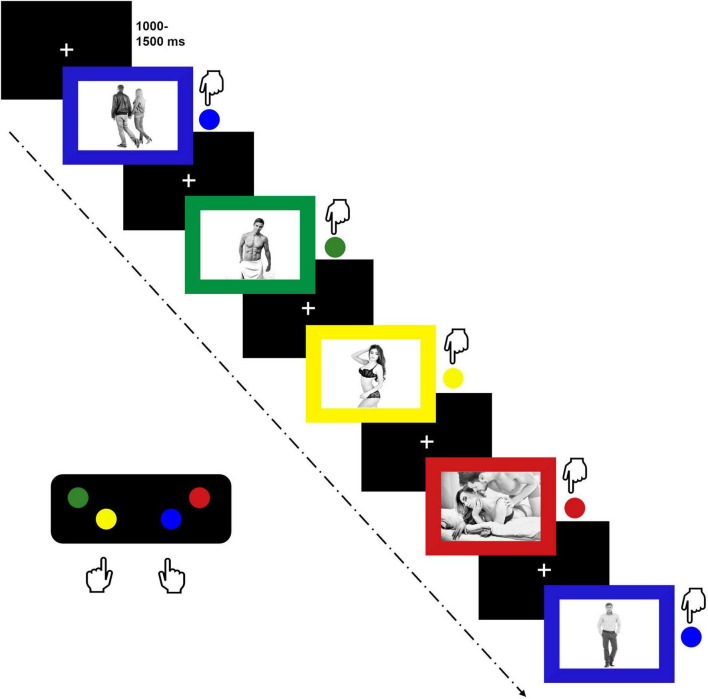
Emotional picture stroop paradigm depicting neutral couples (blue), erotic males (green), erotic females (yellow), erotic couples (red) and neutral persons (second blue).

Stimulus processing was evaluated using an EPSP. This paradigm has proven to be a suitable implicit measure of sexual interest, especially when combined with ERP techniques (Ciardha and Gormley, 2009, 2012; Munk et al., 2018, 2020) and several studies confirm the usability of Emotional Stroop Paradigms for eliciting ERPs (Thomas et al., 2007; Bertsch et al., 2009; Franken et al., 2009; Munk et al., 2018, 2020; Imbir et al., 2021; Paul et al., 2022). Due to the comprehensive nature of the overall project, several emotional stimulus categories were included (Schmidt et al., 2022). However, regarding SE/SI, only the following stimulus categories were considered in the present study: Pictures of scantily dressed erotic couples in intimate poses (couple erotic), pictures of scantily dresses males (male erotic) and females (female erotic) in upright positions, pictures of fully dressed couples (couple neutral) and fully dressed single individuals (person neutral) in neutral poses and neutral facial expressions. Images for the EPSP were obtained from www.shutterstock.com. All pictures were grayscale and displayed in a size of 640 pixels × 480 pixels. Pictures were embedded in colored frames (red, blue, yellow, green). During the task, participants were instructed to indicate the color of the frame by pressing a respective button on a response pad (MiliKey™ MH-5; Lab Hackers Research Equipment, Halifax, Canada). Based on the button arrangement, they were instructed to place their right index and middle fingers on the blue and red buttons and their left index and middle fingers on the yellow and green buttons (see Figure 1). Pictures were presented until participants executed a response and they were instructed to press the respective button as fast and as accurately as possible. Trials were separated using an inter-trial interval of random duration (range 1,000 – 1,500 ms, *M* = 1,250). The color identification task was used to limit conscious evaluative processes related to the erotic stimulus material. Furthermore, it ensured sustained attention to the task, which was of special importance as differences in mind wandering tendencies have been observed between OC using and NC women (Raymond et al., 2019). The EPSP included eight pictures of each stimulus category (indicated in parentheses), presented twice in each of the four colors (red, green, blue, yellow) as illustrated in Figure 1. This resulted in a total of 64 trials per stimulus category. Trials were randomized and divided into four blocks with 30 s breaks in between. Button-arrangement was introduced and recalled using practice trials before each block. The task was presented on a 24” screen using Presentation Software 21.1 (Neurobehavioral Systems Inc. Albany, CA, United States). Participants completed the paradigm in approximately 20 min. More detailed information on the task can be found in Schmidt et al. (2022).

### 2.5 EEG recording

EEG recording was performed using a 64-channel active (Ag/AgCl) electrode system (Brain Products GmbH, Gilching, Germany). Brain Vision software was used for recording (Brain Vision Recorder Version 1.22.0101) and offline processing (Brain Vision Analyzer Version 2.2.0). Signals were digitized using a BrainAmp DC amplifier with a sampling rate of 500 Hz and a band-pass filter from 0.1 to 80 Hz. All sites were re-referenced online to FCz and impedance at each sensor was kept below 20 kΩ. Offline processing started with the application of a 0.5 Hz (12 dB/oct per order) high-pass Butterworth IIR. Data was then visually screened for non-ocular artifacts, and these were subsequently excluded. An Independent Component Analysis (ICA) as implemented in Brain Vision Analyzer was applied to remove blink- and eye-movement artifacts. The resulting data was filtered using a 30 Hz (12 dB/oct per order) low-pass Butterworth IIR and a 50 Hz Notch-filter. After re-referencing to an average reference, EEG epochs were segmented beginning 200 ms before and ending 1000 ms after stimulus presentation. A pre-stimulus interval ranging from −200 to 0 ms was used for baseline correction. LPP amplitudes were quantified as mean amplitudes in the temporal window from 400 to 800 ms. Amplitudes were analyzed at a symmetrical parieto-central electrode cluster including electrodes CP1, CP2, P1, and P2. Time window and electrode sites were chosen based on visual inspection of grand average waveforms and in accordance with prior research (Kuhr et al., 2013; Schindler et al., 2020). To further verify this selection, correlation analyses were conducted to ensure that chosen electrodes form a homogenous cluster of neural activity. Amplitudes at all chosen electrodes correlated significantly with each other (all *p* < 0.001).

### 2.6 Statistical analyses

Statistical analyses were conducted using IBM SPSS Statistics Version 29 (IBM Corp., Somers, NY, United States). Prior to analyses, LPP amplitudes were averaged to receive one score for erotic (averaged over erotic couples, males and females) and one score for neutral stimuli (averaged over neutral single individuals and couples), respectively. For the manipulation check, a repeated measures (rm)ANOVA (within-subjects factor: stimulus category, two steps) was conducted to assess differences between erotic and neutral stimuli regarding LPP amplitudes. As women were assessed in different menstrual cycle/OC regimen phases with possible impact on stimulus processing (Krug et al., 2000; Dreher et al., 2007; Munk et al., 2018, 2020; Schmidt et al., 2022), phase effects regarding the LPP were tested prior to hypothesis testing by computing rmANOVAs separately for each group (between-subjects factors: cycle phase/OC phase, three steps; within-subjects factor: stimulus category, two steps). Regarding FSFI and SE/SI scores, possible associations with relationship status (Wylomanski et al., 2014; Velten et al., 2019b) were tested using two-sample *t*-tests.

Differences between NC and OC women in SE/SI properties were tested using two-sample t-tests.

**FIGURE 2:**
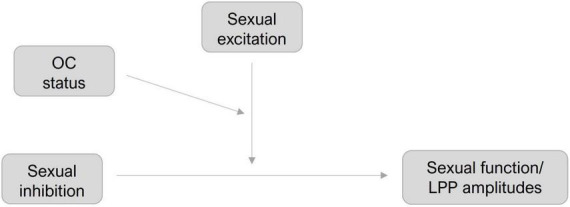
Moderation models for sexual function and LPP amplitudes.

Moderation hypotheses were tested using Hayes model 3 of the SPSS PROCESS Macro (v 4.2) (Hayes, 2018) with a 95% bootstrapping approach (*N* = 5000). SI was set as the predictor and SE and OC-status were added as moderators, respectively (see Figure 2). Self-reported SF (M1) and LPP amplitudes toward erotic (M2) and neutral (M3) stimuli were set as dependent variables, respectively. OC status was effect-coded (−1 = NC, 1 = OC) and SE and SI were mean centered.

## 3 Results

### 3.1 Manipulation check

**FIGURE 3:**
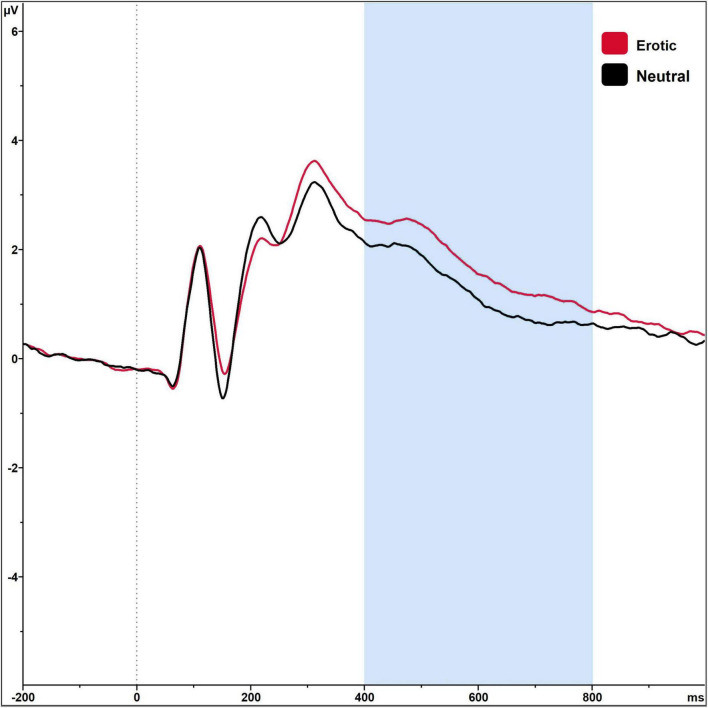
Grand averages on electrode P2 in reaction to erotic and neutral stimuli in *n* = 90 sexually active women in the time window 400–800 ms.

Regarding the LPP amplitudes, there was a significant main effect of stimulus category, *F*_(1,89)_ = 87.52, *p* < 0.001, η_p_^2^ = 0.496, with erotic stimuli eliciting higher LPP amplitudes (μV) compared to neutral ones (Table 1). Grand averages are illustrated in Figure 3. LPP amplitudes did not differ in dependency of menstrual cycle phase or OC regimen phase, all *p* > 0.05. Single women did not differ from women in a committed relationship regarding SE, *t*_(88)_ = −0.82, *p* = 0.412, SI, *t*_(88)_ = 0.61, *p* = 0.547, or the FSFI score, *t*_(88)_ = −0.90, *p* = 0.370.

**TABLE 1:** Descriptive statistics of sexual excitation, sexual inhibition, female sexual function score and mean LPP amplitudes (μV) for the entire sample, naturally cycling (NC) and oral contraceptive using (OC) women.

	Entire sample (*n* = 90)	OC (*n* = 51)	NC (*n* = 39)
**Sexual excitation**			
M ± SD	2.74 ± 0.41	2.63 ± 0.42	2.88 ± 0.35
Range	1.46; 3.83	1.46; 3.46	2.11; 3.83
**Sexual inhibition**			
M ± SD	2.63 ± 0.50	2.72 ± 0.46	2.53 ± 0.53
Range	1.42; 3.75	1.92; 3.75	1.42; 3.47
**Female sexual function index (FSFI) score**			
M ± SD	28.32 ± 4.20	27.61 ± 4.24	29.25 ± 4.02
Range	18.00; 35.40	18.00; 34.60	19.90; 35.40
**Mean LPP amplitudes (in μV) erotic**			
M ± SD	1.46 ± 1.09	1.38 ± 1.08	1.56 ± 1.10
Range	−0.81; 4.36	−0.81; 3.90	−0.41; 4.36
**Mean LPP amplitudes (in μV) neutral**			
M ± SD	0.98 ± 1.07	0.91 ± 1.06	1.07 ± 1.08
Range	−1.50; 3.65	−1.50; 3.55	−0.97; 3.65

### 3.2 Sexual excitation and inhibition in dependency of OC use

Women using OCs reported significantly lower SE as compared to NC women, *t*_(88)_ = 3.03, *p* = 0.003, *d* = 0.65, but did not differ regarding SI, *t*_(88)_ = −1.81, *p* = 0.074. Descriptive statistics are presented in Table 1.

### 3.3 Sexual function in dependency of sexual excitation, sexual inhibition and OC use

**FIGURE 4:**
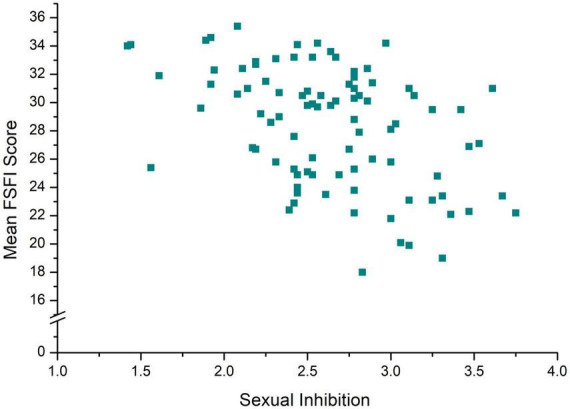
Mean female sexual function index (FSFI) score in dependency of sexual inhibition of *n* = 90 sexually active women.

Regarding SF, the moderation model, *F*_(7, 82)_ = 3.62, *p* = 0.002, *R*^2^ = 0.24 (see Table 2), revealed a significant negative association between SI and FSFI scores as illustrated in Figure 4. Neither SE, nor the SE × SI Interaction or any OC-related interaction term were statistically significant.

**TABLE 2:** Moderation analysis for the female sexual function index (FSFI) total score (Hayes Model 3).

	*b* [LLCI, ULCI]	SE	*t*	*p*
constant	28.27 [27.37, 29.16]	0.46	64.74	<0.001***
SI	−3.27 [−5.16, −1.01]	1.05	−3.77	<0.001***
SE	1.20 [−1.28, 3.76]	1.28	1.06	0.291
SI × SE	−1.25 [−8.26, 3.02]	2.84	−0.23	0.818
OC	−0.37 [−1.26, 0.53]	0.46	−0.76	0.449
SI × OC	−0.47 [−2.78, 1.55]	1.07	−0.45	0.657
SE × OC	−1.07 [−3.52, 1.48]	1.28	−0.99	0.323
SI × SE × OC	0.13 [−4.32, 7.27]	2.84	−0.26	0.799

***p < 0.001. LLCI, lower limit confidence interval; ULCI, upper limit confidence interval; SI, sexual inhibition; SE, sexual excitation; OC, oral contraception status.

### 3.4 LPP amplitudes toward erotic stimuli in dependency of sexual excitation, sexual inhibition and OC use

**FIGURE 5:**
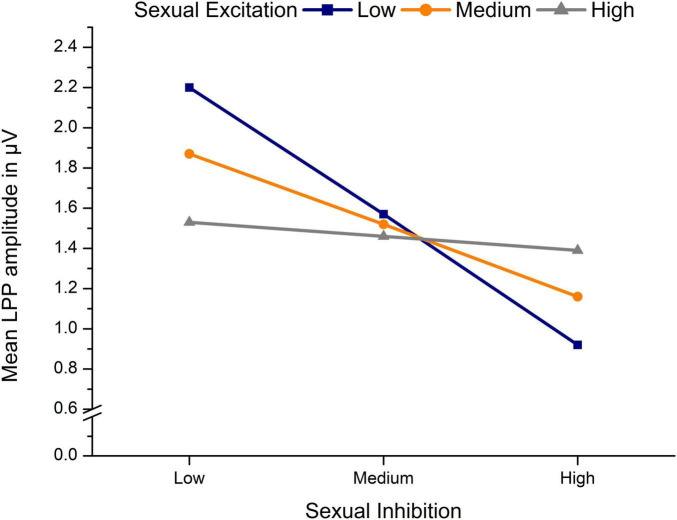
Mean LPP amplitudes in μV toward erotic stimuli in dependency of sexual inhibition and sexual excitation of *n* = 90 sexually active women.

The significant moderation model regarding LPP amplitudes toward erotic stimuli, *F*_(7,82)_ = 2.33, *p* = 0.032, *R*^2^ = 0.17 (see Table 3), indicated a significant effect of SI as well as a significant SE × SI interaction. The three-way interaction was not significant indicating that the size of the SE × SI interaction did not differ significantly between OC using and NC women. The significant SE × SI interaction indicated that, in both, NC and OC women, SE and SI interacted to predict LPP amplitudes. Simple slope analysis showed that negative effects of SI on LPP amplitudes were significant only among low, *t*_(86)_ = −3.96, *b* = −1.30, *p* < 0.001, and medium, *t*_(86)_ = −3.20, *b* = −0.72, *p* = 0.002, but not among high levels of SE, *t*_(86)_ = −0.54, *b* = −0.15, *p* = 0.592, (see Figure 5).

**TABLE 3:** Moderation analysis for the LPP amplitudes toward erotic stimuli (Hayes Model 3).

	*b* [LLCI, ULCI]	SE	*t*	*p*
constant	1.54 [1.33, 1.76]	0.11	12.96	<0.001***
SI	−0.71 [−1.21, −0.17]	0.27	−2.78	0.007**
SE	−0.16 [−0.76, 0.43]	0.30	−0.49	0.626
SI × SE	1.32 [0.20, 2.42]	0.56	2.47	0.016*
OC	−0.09 [−0.31, 0.12]	0.11	−0.69	0.494
SI × OC	−0.16 [−0.73, 0.34]	0.27	−0.63	0.529
SE × OC	−0.04 [−0.64, 0.57]	0.30	−0.22	0.830
SI × SE × OC	−0.15 [−1.28, 0.88]	0.54	−0.13	0.894

*p < 0.05,

**p < 0.01,

***p < 0.001. LLCI, lower limit confidence interval; ULCI, upper limit confidence interval; SI, sexual inhibition; SE, sexual excitation; OC, oral contraception status.

### 3.5 LPP amplitudes toward neutral stimuli in dependency of sexual excitation, sexual inhibition and OC use

Regarding LPP amplitudes toward neutral stimuli, the moderation model was not significant, *F*_(7,82)_ = 1.72, *p* = 0.116, indicating that neither SE/SI nor OC status or respective interaction terms significantly predicted LPP amplitudes toward neutral stimuli.

## 4 Discussion

### 4.1 General discussion

The primary objective of this study was to investigate the interactive effects of SE and SI on self-reported SF and the neural processing of erotic stimuli. In addition, potential effects of OC use on SE/SI properties and their dynamic interaction were evaluated. Regarding self-reported SF, results revealed a negative association with SI levels, without significant relationships regarding SE or SE/SI interactions. However, when examining LPP amplitudes toward erotic stimuli, SE/SI interactions emerged, with the diminishing effects of SI on LPPs being attenuated among high SE levels. Moreover, the findings indicated lower SE in OC users compared to NC women with no significant differences observed in SI levels.

#### 4.1.1 Sexual function

In the current stury, women with stronger SI properties reported lower SF. This is in line with previous studies reporting a prominent role of SI regarding sexual dysfunctions (Moura et al., 2020; Quinta-Gomes et al., 2022) and negative associations between SI and genital arousal (Janssen et al., 2002b; Hodgson et al., 2016). Highly inhibited individuals exhibit greater sensitivity to dampening situational cues, such as relationship insecurity, or performance concerns. Consequently, they might feel insecure with a partner, experience excessive worry or anxiety during sexual intercourse and find it difficult to relax. This might result in reduced SF and even sexual dysfunction such as HSDD, or orgasmic and genito-pelvic pain disorder (Payne et al., 2005; Moura et al., 2020). In turn, however, prior experience of sexual dysfunctions, such as struggling to maintain arousal or to reach an orgasm, might lead to increased self-monitoring and worrying during sexual intercourse, contributing to heightened self-reported SI. In contrast to earlier research (Janssen et al., 2002b; Clifton et al., 2015; Hodgson et al., 2016; Velten et al., 2019a), no associations between SF and SE were observed in the current sample. Differential analytic techniques, i.e., correlation vs. multiple regression, might underlie these inconsistencies. Furthermore, SE properties might be more strongly associated with specific facets of SF (Nolet et al., 2021). Whereas SE dynamics (i.e., being aroused or “turned-on”) are mainly represented in the desire domain, SI items demonstrate strong content-related overlap with the FSFI domains arousal, lubrication, and orgasm. This could explain the higher relevance of SI regarding SF. We, therefore, exploratorily conducted a moderation analysis regarding the FSFI desire domain. Results showed that SE was positively associated with desire whereas a negative association emerged regarding SI. As for the total score, no interactive effects were observed.

#### 4.1.2 Neural correlates of sexual excitation and inhibition

Contrary to the results concerning SF, a significant SE/SI interaction effect emerged regarding measured LPP amplitudes. Higher SI was associated with dampened LPP amplitudes toward erotic stimuli, this association was, however, attenuated among high SE levels. This suggests that strong excitatory properties might help individuals to overcome inhibition. The implications of this finding are twofold. Firstly, the observed SE/SI interaction supports the theoretical assumptions of the DCM framework (Janssen and Bancroft, 2023), that predicts interactive as opposed to independent SE/SI effects. This might also clarify why inconsistencies regarding SE/SI effects were observed in prior research (Prause et al., 2008; Carvalho et al., 2018). While most previous studies did not consider interactive effects, a significant interaction has also been noted in a study by Velten et al. (2016c) reporting a positive association between SE and genital arousal among low levels of SI but a negative association among high SI levels. The authors concluded that inner conflicts resulting from high levels of both forces could underlie this interference effect. Conversely, our results suggest a compensation effect in that high SE levels buffer diminishing effects of high SI. The differential pattern compared to the Velten et al. (2016c) study could be attributed to the different outcome measures (genital arousal vs. neural reactivity) and the invasive procedure required to assess genital arousal, i.e., by means of a vaginal photoplethysmography. This might be especially uncomfortable for highly inhibited individuals and could explain their decreasing genital arousal with increasing SE. Neural reactivity is, however, less likely impacted by shame or social desirability as it cannot be consciously perceived or influenced and is measured non-invasively.

Secondly, the results point toward an important role of inhibitory mechanisms in erotic stimulus processing. This is crucial, as most previous studies focused solely on the role of excitatory properties in attending to and processing of erotic stimuli (Prause et al., 2008; Carvalho et al., 2018; Janssen and Bancroft, 2023). However, previous research had already indicated that SI is equally relevant regarding stimulus processing. Turner et al. (2019), for example, reported higher stimulus avoidance regarding erotic stimuli in high SI individuals. This finding is in line with the lower LPP amplitudes observed in the current study. Aguiar et al. (2023), however, observed heightened initial, automatic attention to erotic stimuli, as measured using N200 amplitudes, in association with high SI. While these findings seem contradictory at first, they not necessarily are. Inhibitory processes are necessary to suppress sexual responses in circumstances where they could be inappropriate or even dangerous. Highly inhibited individuals might, therefore, demonstrate stronger associations between sexual cues and feelings of danger, worry and anxiety. Indeed, individuals with sexual dysfunctions express more negative automatic thoughts regarding/during sexual activity (Tavares et al., 2020). Due to these threatening associations regarding sexual cues, highly inhibited individuals might display heightened vigilance to such cues, resulting in increased initial processing (Aguiar et al., 2023). As a result of this increased early awareness of erotic content, highly inhibited individuals might experience a strong suppression of approach behavior toward erotic stimuli as observed in the negative association between SI and LPP amplitudes. As the current study focused on motivational rather than early attentional mechanisms associated with SE/SI, the N200 component was not considered. This component is usually examined within specific experimental tasks, such as the oddball task used by Aguiar et al. (2023), flanker tasks or go/no-go tasks eliciting response conflict or response inhibition (Folstein and van Petten, 2008). While such effects can be observed in “traditional” Stroop Tasks, Thomas et al. (2007) have previously shown that Emotional Stroop Paradigms are unlikely to elicit relevant N200 effects. Nevertheless, it is important to note that both early as well as later stages of stimulus processing seem to be impacted by propensities for SI.

Furthermore, Aguiar et al. (2023) observed a positive association between SI and N200 amplitudes in response to erotic as well as to romantic stimuli which depicted dressed couples in intimate situations, but not engaging in sexual intercourse. In the current study, the overall moderation model regarding neutral stimuli was non-significant. However, we visually noted a SE/SI interaction that was similar to that observed regarding erotic stimuli. That is, LPP amplitudes decreased with increasing SI, but this effect was attenuated among high SE levels. One possible explanation for this pattern is that erotic stimuli are also inherently social stimuli. Correspondingly, SE/SI scales overlap with behavioral activation (BAS) and behavioral inhibition (BIS) scales that describe general approach and avoidance tendencies (Rettenberger et al., 2016; Bártová et al., 2021). Moreover, sexual dysfunctions, which can also be predicted by SE/SI properties, are associated with social anxiety (Figueira et al., 2001; Bodinger et al., 2002). Individuals high in SI might experience a general tendency toward social inhibition and social insecurity, as indicated by their fear of negative judgment regarding sexual activity or performance. It is, therefore, reasonable to expect associations of SE/SI with processing of non-sexual social stimuli as well.

For low SI levels, the current results suggest relatively lower neural reactivity for women high vs. low in SE. In accordance with Hodgson et al. (2016) and Prause et al. (2008), this could be interpreted as habituation or boredom effects resulting from increased exposure of this group to sexual stimulus material.

#### 4.1.3 Associations of oral contraception use with sexual excitation and inhibition properties

To the best of our knowledge, effects of OC use on SE/SI properties have not previously been studied, therefore, our findings are especially important. They suggest an association between OC use and attenuated excitatory properties, aligning with preceding studies reporting decreased sexual desire in OC users (Zethraeus et al., 2016; Huang et al., 2020). A reduction in free testosterone, a gonadal hormone which is relevant regarding sexual motivation (Wu et al., 2022), is suggested to underlie such effects, although supporting evidence is inconclusive (Graham et al., 2007; Zethraeus et al., 2016). Earlier research has also indicated reduced SF and erotic stimulus processing in OC users (Wallwiener et al., 2010; Abler et al., 2013; Monciunskaite et al., 2019). However, our moderation models did not yield significant OC effects, consistent with the mixed results in this field (Schaffir, 2006; Schmidt et al., 2022). These inconsistencies highlight the importance of considering psychosexual variables such as SE/SI in sexuality research alongside biological predictors like sex steroid concentration or OC use. They, furthermore, pose the question if SE/SI properties undergo changes upon initiating OC use, or if, in turn, SE/SI properties might influence contraceptive choices. A reduction of SE following OC initiation could be a plausible explanation. However, our results revealed a statistical trend (*p* = 0.074) toward higher SI in OC users compared to NC women. While it is rather unlikely that low excitation triggers the choice of OCs as a contraceptive method, heightened inhibition might drive the selection of a highly effective contraceptive option, empowering women to feel in control of their reproductive choices. The initiation of OC use could then potentially lead to reduced sexual excitability. However, elevated SI levels could also be a secondary outcome of reduced excitability following OC use as the loss of libido might result in interpersonal conflict in relationships or discomfort during intercourse (Géonet et al., 2013; Willoughby et al., 2014). It is crucial to note that these proposed associations remain speculative, and longitudinal studies are needed to explore possible developmental trajectories.

Moreover, observed OC effects are also relevant regarding reported gender differences in SE/SI with typically higher SE in males compared to females (Carpenter et al., 2008; Granados et al., 2020b). These established gender-based patterns should be re-evaluated taking into account the potential influence of hormonal contraception.

### 4.2 Implications and future directions

To the best of our knowledge, a hypervigilance-avoidance pattern, as suggested by increased N200 (Aguiar et al., 2023) and reduced LPP amplitudes observed in our study, has not been systematically examined in association with SE/SI or sexual dysfunctions. It is, however, common in anxiety disorders, such as social anxiety (Bögels and Mansell, 2004; Vassilopoulos, 2005), which have a high comorbidity with sexual dysfunctions (van Lankveld and Grotjohann, 2000; Figueira et al., 2001; Laurent and Simons, 2009). Future research should, therefore, investigate associations between SE/SI or sexual dysfunctions and distinct stages of erotic stimulus processing in order to reveal underlying patterns.

Reported results also have implications for the development of treatment options for sexual problems. This is especially important, since sexual dysfunctions are common, whereas available treatment options are still sparse, specifically for women (McCabe et al., 2016; Weinberger et al., 2019). In recent years, there is increasing awareness that, within the DCM framework, high SI underlying sexual dysfunctions could require differential treatment as opposed to low SE. Correspondingly, Poels et al. (2014) tested distinctive drug treatments for HSDD depending on the strength of excitatory vs. inhibitory properties. Women with low SE received testosterone in combination with a phosphodiesterase type 5 (PDE-5) inhibitor. Women high in SI were treated with a combination of testosterone, to increase sexual motivation, and a 5HT_1A_ receptor agonist, to reduce inhibition. Both treatments showed beneficial effects regarding SF (see also Bancroft et al., 2009; Bloemers et al., 2013; van Rooij et al., 2013). Our results strengthen the notion that a combined approach, simultaneously fostering sexual motivation and reducing inhibition, might be advantageous for highly inhibited individuals. Studies assessing associations between SE/SI and individual differences in endogenous sex steroid (i.e., estrogen, progesterone, testosterone) or neurotransmitter (i.e., dopamine, serotonin) function could be relevant in identifying possible vulnerability factors for sexual dysfunctions or risky sexual behavior and corresponding treatment approaches (Clayton, 2010; Kühn and Gallinat, 2016).

With regard to psychotherapeutic techniques, mindfulness interventions have been proven effective in improving SF (Silverstein et al., 2011; Milani et al., 2021). They could aid in decreasing mind-wandering and worrying during sexual intercourse and thus ultimately reduce inhibition. They could be paired with interventions strengthening positive and rewarding associations with erotic stimuli such as cognitive restructuring, or sensate focus in accordance with Masters and Johnson (Masters and Johnson, 1970; Leiblum and Wiegel, 2002; Brauer et al., 2012). For development of such interventions, studies assessing associations of SE/SI with sexual cognitions, including positive or negative automatic thoughts during intercourse (Tavares et al., 2020), would be insightful.

Regarding pharmacological as well as psychotherapeutic interventions, ERP techniques could be a valuable approach to evaluate treatment success, as they are less impacted by social desirability, or other confounding factors, such as shame, compared to self-reports or genital measures. They could also be used to identify specific mechanisms of action and, thus, improve treatment options for affected persons.

Concerning methodological considerations, future studies on SE/SI would benefit from incorporating eye-tracking measures. Former studies in individuals with sexual dysfunctions noted differential viewing patterns of erotic scenes, i.e., greater focus on background or context details compared to sexually explicit stimulus features (Lykins et al., 2011; Velten et al., 2021). Correspondingly, individuals with different SE/SI properties might focus on dissimilar stimulus features, although this has not previously been examined. As eye movement patterns were not assessed in the current study, we cannot rule out that differential viewing patterns underlie reported results. Furthermore, interactive effects of SE/SI might be more pronounced when used stimuli depict aspects of sexual interactions directly related to SI (e.g., pain, judgement, risk of pregnancy/STDs), or SE (e.g., unusual settings, dominance). Hence, using a greater variety of sexuality-related stimulus material (positive, negative, high vs. low in context details), or more explicit stimuli depicting different stages of sexual readiness (Huberman et al., 2023), would be insightful. In this regard, implicit measures of stimulus processing could be paired with explicit stimulus evaluations (i.e., valence and arousal ratings). Such ratings were collected in the current study, however, we they were not included in the present analysis. As part of an extensive research project, dealing with effects of menstrual cycle/OC regimen phases, women were assessed in three different cycle phases. To exclude effects of conscious stimulus evaluation on stimulus processing, ratings were collected only on the third measurement occasion. Therefore, ratings were not included in the present analyses due to the different measurement occasions on which ERPs vs. ratings were collected (T1 vs. T3). Furthermore, the fact that stimuli were only rated after repeated exposure could also have affected the stimulus evaluations, especially for subjects prone to SE who might show stronger boredom effects. Further studies are, therefore, needed to examine the role of interactive SE/SI effects in predicting subjective stimulus evaluations.

### 4.3 Limitations

The current study is among the first to examine the DCM using ERP techniques, offering valuable insights into the potential association between altered stimulus processing and SE/SI in the context of sexual dysfunctions. Nevertheless, some limitations have to be considered when interpreting and applying reported results.

Primarily, an exclusively female sample was analyzed. Given the differing social stigmas and norms associated with male and female sexuality (Emmerink et al., 2016), typical gender differences in SE and SI, where males tend to exhibit higher SE and lower SI compared to females (Carpenter et al., 2008; Granados et al., 2020b), as well as evidence suggesting that SE and SI relate differently to sexual behavior in males and females (Granados et al., 2020b), it is essential to recognize that SE and SI may have distinct implications in males as opposed to females.

Moreover, the present study focused on a healthy sample, without any physical or psychological health condition, including diagnosed sexual dysfunction. It is important to consider that results in clinical samples might differ and necessitate additional research. However, it is noteworthy that around one third of our sample scored below the clinical cutoff score of 26.55 on the FSFI (Wiegel et al., 2005), indicating potential similarities with findings in clinical samples. Furthermore, analyses were limited to sexually active women to avoid confounding of OC status and sexual activity (due to the higher proportion of sexually active women in the OC group). This sample selection allowed to distinguish OC effects on SE/SI properties, sexual function and LPP amplitudes from those related to sexual activity but resulted in a decreased overall sample size and unequal sample sizes of the NC and OC groups. Thus, groups might have been too small to detect OC related differences in SE/SI dynamics. Furthermore, women reporting no sexual activity within the last 4 weeks reported significantly higher levels of SI compared to sexually active women, therefore, the dynamic interplay between SE/SI might be shifted toward inhibition. We, therefore, exploratively conducted moderation analyses separately in the sexually inactive women. The overall model was not significant (possibly due to the small sample size of *N* = 24). Visually, however, we observed a SE/SI interaction pattern that was qualitatively different from that observed in the sexually active women. In sexually active women, LPP amplitudes decreased with increasing SI and this effect was attenuated among high SE levels. In sexually inactive women, LPP amplitudes decreased with increasing SI but only in those low in SE. In women high in SE, LPP amplitudes slightly increased with increasing SI. Due to the small sample size, these results should be interpreted with caution. They do, however, suggest that erotic stimulus processing could be influenced by sexual activity. In future studies, larger samples are required to further assess the possible impact of OC use and sexual activity on SE/SI dynamics. These should ideally include equal proportions of sexually active and inactive women (using or not using hormonal contraception) because research comparing erotic stimulus processing in sexually active vs. inactive individuals is generally scarce.

Additionally, it is important to note that the SESII-W scale for females (Graham et al., 2006; Velten et al., 2016a) was used in the current study, whereas Aguiar et al. (2023) used the SIS/SES scales that are applicable for male as well as female samples (Janssen et al., 2002a). The SESII-W was chosen in the current study because it was specifically designed to assess aspects relevant for female sexual responses and also because it has been widely used in studies focusing on exclusively female samples (Clifton et al., 2015; Velten et al., 2016c; Granados et al., 2020a). Compared to the SIS/SES-SF, the SESII-W also demonstrates better psychometric properties in female samples (Hodgson et al., 2016; Velten et al., 2016a; Rettenberger et al., 2019). The SIS/SES scales, however, differentiate two aspects of SI further, namely a SIS1 factor that assesses SI due to threat of performance failure and a SIS2 factor assessing SI due to threat of performance consequences. Use of this scale might, therefore, be beneficial in evaluating the specific aspects of SI that are associated with stimulus processing.

## 5 Conclusion

The current findings highlight the importance of interactive SE/SI effects, providing additional psychophysiological evidence supporting the theoretical assumptions of the DCM. They are, furthermore, also relevant regarding the understanding of female sexuality and related dysfunctions. Reported findings may guide the development of targeted psychological interventions addressing such dysfunctions. While sexual dysfunctions seem to be primarily predicted by high SI, individuals might still benefit from therapeutic interventions that strengthen excitatory forces. The association of OC use with diminished SE properties is noteworthy and might have implications for managing low sexual desire and sexual dysfunctions in OC users. Additionally, observed group differences in SE are also relevant regarding reported gender differences in SE/SI properties. Collectively, these findings enhance our understanding of the dynamic interplay between SE and SI and their implications for psychological interventions in the context of female sexuality.

## Data availability statement

The raw data supporting the conclusions of this article will be made available by the authors (JH), without undue reservation.

## Ethics statement

The studies involving humans were approved by the Local Ethic Commission of the Faculty of Psychology and Sport Science at Justus-Liebig-University Giessen, Germany. The studies were conducted in accordance with the local legislation and institutional requirements. The participants provided their written informed consent to participate in this study.

## Author contributions

NS: Conceptualization, Data curation, Formal analysis, Investigation, Methodology, Visualization, Writing – original draft, Writing – review and editing. JH: Conceptualization, Methodology, Resources, Software, Supervision, Writing – review and editing. AM: Conceptualization, Funding acquisition, Methodology, Project Administration, Resources, Software, Supervision, Writing – review and editing.
